# Maternal patterns of inheritance alter transcript expression in eggs

**DOI:** 10.1186/s12864-023-09291-8

**Published:** 2023-04-10

**Authors:** Nathan D. Harry, Christina Zakas

**Affiliations:** Department of Biological Sciences, North Carolina State University, 112 Derieux Place, Raleigh, NC 27607 USA

**Keywords:** Maternal effects; regulatory evolution; EvoDevo; parental genetic effects

## Abstract

**Background:**

Modifications to early development can lead to evolutionary diversification. The early stages of development are under maternal control, as mothers produce eggs loaded with nutrients, proteins and mRNAs that direct early embryogenesis. Maternally provided mRNAs are the only expressed genes in initial stages of development and are tightly regulated. Differences in maternal mRNA provisioning could lead to phenotypic changes in embryogenesis and ultimately evolutionary changes in development. However, the extent that maternal mRNA expression in eggs can vary is unknown for most developmental models. Here, we use a species with dimorphic development— where females make eggs and larvae of different sizes and life-history modes—to investigate the extent of variation in maternal mRNA provisioning to the egg.

**Results:**

We find that there is significant variation in gene expression across eggs of different development modes, and that there are both qualitative and quantitative differences in mRNA expression. We separate *parental* effects from *allelic* effects, and find that both mechanisms contribute to mRNA expression differences. We also find that offspring of intraspecific crosses differentially provision their eggs based on the parental cross direction (a *parental* effect*)*, which has not been previously demonstrated in reproductive traits like oogenesis.

**Conclusion:**

We find that maternally controlled initiation of development is functionally distinct between eggs of different sizes and maternal genotypes. Both allele-specific effects and parent-of-origin effects contribute to gene expression differences in eggs. The latter indicates an intergenerational effect where a parent’s genotype can affect gene expression in an egg made by the next generation.

**Supplementary Information:**

The online version contains supplementary material available at 10.1186/s12864-023-09291-8.

## Background

The unfertilized egg is a key time point to understand how differences in maternal provisioning affect ontogeny. In all metazoans, the first stages of embryonic development are carried out entirely by maternal proteins and mRNAs loaded into the oocyte [1–3]. Control of development only transfers to the zygotic genome after a few cell divisions during a period called the maternal-to-zygotic transition, when the zygotic genome is activated by maternal transcription factors [1–5]. Because maternal transcripts directly control the offspring’s initial development, variation in maternal mRNA composition and abundance could profoundly alter subsequent developmental processes [6–9]. Changes in maternal mRNA expression may allow for shifts in development that ultimately lead to changes in life-history traits [10–14].

Egg size is a cornerstone trait of life-history theory and developmental biology. Variation in egg size, even in offspring with the same zygotic genotypes, can alter life-history traits [15, 16]. However, while egg size is often used as a proxy for maternal investment, size and maternal mRNA investment may not always scale [17–21]. So how are egg size and maternal mRNA deposition related? There are some examples where egg size is linked with differences in maternal RNA deposition: there is a change in growth-related gene expression with different egg sizes across cichlid species [22]. In frogs maternal RNA localization differs in species with different egg sizes [23, 24] and RNA content changes with naturally occurring egg size differences within species [25]. However, it remains unknown whether mRNA expression will scale with increased egg size (quantitative change), or if completely different mRNAs are provided in conjunction with metabolic demands or new cellular functions (qualitative change).

### Study system

**Fig. 1:**
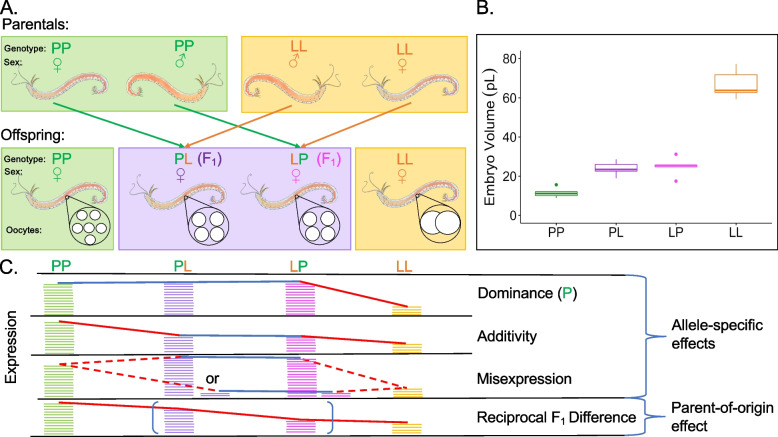
**A** Top: Reciprocal cross schematic to generate F_1_ females. Bottom: Representative females and eggs generated from crosses. **B** Early embryo area as a proxy for egg size in the four categories of females used in this study. F_1_ females have intermediate egg sizes compared to the parental types. Number of clutches measured for PP = 6, LL = 5, PL = 8, LP = 4 (10 embryos measured per clutch) Quantiles are shown with box-plots with dots representing outliers. **C** Allele-specific expression is when parental strains have differential expression, and PL and LP have matching expression levels relative to each other. Colored, horizontal lines represent mRNA abundance in the indicated groups, with fewer lines representing reduced gene expression for a given gene. A parent-of-origin effect is when the reciprocal F_1_s have differing expression levels relative to each other and the parentals. Blue lines indicate the same expression levels and red lines indicate a decrease in expression. Misexpression can be either over or underdominance

To investigate how mRNAs are provisioned to eggs of different sizes (and consequent development modes), we use the marine annelid *Streblospio benedicti*. This species exhibits a developmental dimorphism: there are two types of females found in natural populations that produce eggs of different sizes: 100 μm and 200 μm diameter eggs that have an eight-fold difference in volume (Fig. 1b). The offspring develop into different larval types with alternate life-histories categorized by their trophic mode. Small eggs develop into planktotrophic (PP) larvae (obligately plankton feeding) and large eggs develop into lecithotrophic (LL) larvae (yolk-feeding; [26, 27]). PP mothers produce hundreds of small eggs per clutch and the larvae develop a gut and swimming structures early in development [28, 29]. By comparison, LL mothers produce tens of larvae per clutch with no swimming structures. LL larvae have an abbreviated larval phase, different larval morphologies, and require only maternally provided energy to undergo metamorphosis [30]. Despite these developmental differences, both larval morphs converge on the same body plan after metamorphosis. This intraspecific developmental dimorphism provides an opportunity to study how development can evolve from differences in egg provisioning.

Previous work in *S. benedicti* shows the genetic basis of alternative larval phenotypes is modular, with independent loci affecting individual traits [31]. For egg size in particular, there are both parental and zygotic loci that affect size [16]. However, the parental loci can act in both paternal and maternal effect directions [32]. This means that egg size is partially determined by inherited alleles, but also by the genotype of its parents. The mechanism by which these parental effects shape development and egg size is unknown.

As a single species, adults that arise from the two larval types of *S. benedicti* can be crossed to produce viable F_1_ offspring. Crosses can be reciprocal, alternating parental roles between PP and LL adults. F_1_ females produce eggs of intermediate size compared to their parents, which will develop into F_2_ offspring with segregating larval traits ([16, 31]; Fig. 1). This allows us to use F_1_ females’ eggs to disentangle the effects of egg size and mRNA expression.

### Approach

In this study we use *S. benedicti* to understand how maternally provided transcripts are associated with egg size. We determine if a difference in egg size is correlated with a quantitative change in mRNA provisioning by comparing gene expression in the eggs. While there is a large difference in overall egg size between the morphs, we do not expect there to be a difference in the total amount of RNA expressed in the two types of eggs. Much of the difference in volume of the eggs is due to yolk and lipid deposits, with the LL eggs having far more yolk [33]. When extracting total mRNA from eggs, we did not see a difference in quantifiable yield between oocytes (data not shown). This means that while there are differences in egg size, there should not be a difference in overall amount of mRNA per egg. We ask if changes in egg size are accompanied by mRNA expression differences, not because larger eggs might have more RNA, but because reproductive changes in oogenesis that lead to larger eggs may have pleiotropic effects on gene expression levels.

We use the eggs of F_1_ females from crosses between the two developmental types to investigate the regulatory architecture of mRNA expression changes. Using F_1_ females’ eggs allows us to disentangle allele-specific effects on expression from parent-of origin effects (Fig. 1c). For allele-specific expression, we determine the mode of inheritance for differentially expressed genes and show the extent of allelic dominance and additivity. In contrast, we determine the effects of parent-of-origin: where the expression level depends on if the allele originated from the mother or father. Parent-of-origin differences in gene expression between reciprocal F_1_s indicate an intergenerational effect on oocyte mRNA provisioning; isolating allele-specific and parent-of-origin inheritance patterns allows us to understand the extent to which these mechanisms contribute to variation in mRNA expression in eggs. However, this analysis is only possible in species where reciprocal crosses are viable, which is rare when considering crosses of individuals with dramatically different egg sizes and developmental modes. In fact, no studies to date have demonstrated an intergenerational effect on egg mRNA provisioning.

By using eggs of F_1_ mothers we can determine the regulatory architecture of differentially expressed genes: expression differences can be attributed to *cis* or *trans*-acting genetic factors (reviewed in [34]). Allele-specific differences in hybrids or strain-crosses have been used to investigate regulatory changes in a number of models: yeast [35–37], flies [38–42], plants [43, 44], fishes [45, 46], and mice [47, 48]. Such studies have demonstrated that gene regulatory differences between species can be predominantly driven by either *cis* or *trans*-acting factors, and this varies depending on the species crossed. Studies in *Drosophila* that account for evolutionary divergence times across species show that *cis*-acting factors are greater in interspecific hybrids than for intraspecific F_1_s [41]. We test for the effects of *cis* and *trans* regulatory modifications on differentially expressed genes in the eggs made by F_1_ mothers (the eggs that would give rise to the F_2_ generation). Therefore, we can determine if mRNA expression differences are due to *trans or cis*-acting regulatory elements in the maternal genome. As *S. benedicti* is a single species with little genome-wide differentiation [31, 49], this analysis shows how genetic divergence of coupled reproductive and life-history transitions may first begin to evolve.

## Results

### Comparison of LL and PP egg gene expression

We dissected the unfertilized oocytes from PP and LL females and used pooled oocytes to make mRNA libraries (Fig. 1a). Both sets of libraries were aligned to the *S. benedicti* reference genome for differential expression analysis (summary shown in STable 1). Total mRNA differences between the libraries will not change the significance of differential expression, as that is accounted for in the normalization. Therefore no effect of potential total RNA differences on expression is observed in the results because they are based on relative RNA abundance. For example, we did not observe differential expression in housekeeping genes between the two morphs. Our criteria for differential expression is that genes have a 1.5 fold change in expression value, and a multiple-test corrected (Benjamini-Hochberg) *p*-value of less than 0.05.

**Fig. 2:**
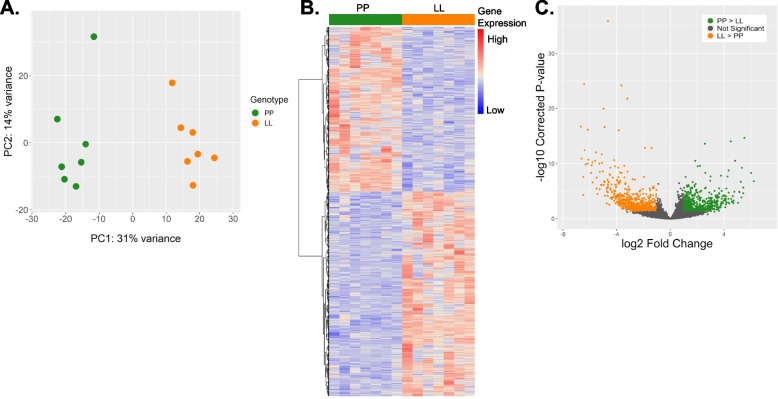
Differential Gene expression between PP and LL oocytes. **A** PCA showing distinct clustering of samples by genotype on PC1. **B** Heatmap of differentially expressed genes between PP and LL samples showing relative gene expression. Colors are scaled per-gene. **C** Volcano plot showing genes upregulated in LL samples (orange) and genes upregulated in PP samples (green) with their log2-fold expression change between morphs and multiple-test corrected *p*-values

There are many genes that are significantly differentially expressed (Wald test with DESeq2; [50]) between the eggs of the two morphs (1,155 genes) and the principal component analysis (PCA) indicate that we capture significant biological differences between groups; the samples separate by developmental mode on the first principal component (Fig. 2a). While it is clear that there is significant within-group variation in oocyte mRNA provisioning, shown on PC2 (14% variance), more than twice that variation is found between groups (PC1, 31% variance). The differentially expressed genes make up 10.24% of total expressed genes (*n* = 11,161 genes) and 4.4% of the total number of genes in the *S. benedicti* reference genome (*n* = 26,216 genes). 515 genes (44.6% of the differentially expressed genes) are expressed more in PP oocytes, and 640 are expressed more in the LL oocytes (Fig. 2b, c). Of those 1,155 differentially provisioned genes, 92 genes (8%) are non-coding RNAs.

Genes that are only expressed in one of the morphs are considered exclusive. 18 genes have very low (sample group mean read count is fewer than 10 reads) or no expression in one morph. The PP eggs express eight exclusive genes and the LL eggs express ten exclusive genes. Most of these genes are unknown in function (STable 2).

Examination of the functional gene annotations of differentially expressed genes reveals several potentially interesting avenues for further study: Zinc finger proteins account for 12% (144 genes) of differentially expressed genes and often serve transcriptional regulators. Because of the relatively low gene annotation rate in this dataset and lack of spiralian-specific genes in most commonly used gene databases, it is possible that such proteins may be lineage specific transcription factors, but this requires further functional validation. We also find that many known transcription factors are differentially expressed between PP and LL oocytes, including forkhead box protein (Sbene_G06980), polycomb protein (Sbene_G10227), kruppel-like factor 15 (Sbene_G10036), visual system homeobox 2 (Sbene_G05917), and paired-box protein (Sbene_G17489). Additionally, we identify genes involved in the Wnt signaling pathway, such as frizzled-5 (Sbene_G05398) and six putative copies of Notch (Sbene_G06517, Sbene_G09676, Sbene_G09677, Sbene_G13608, Sbene_G15020, Sbene_G18208; Additional annotations in SFig. 1).

### Comparison of reciprocal F_1_ gene expression with LL and PP

**Fig. 3:**
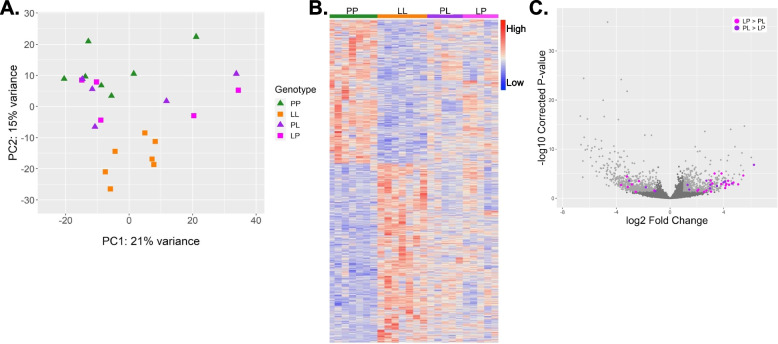
Differential gene expression between PL and LP oocytes. **A** PCA shows F_1_ expression is intermediate to the parentals. **B** Heatmap shows both directions of F_1_’s relative gene expression alongside PP and LL expression. Colors are scaled per-gene. **C** Volcano plot of differential expression between P and L with 38 genes that are also significantly differentially expressed between F_1_ crosses highlighted in pink or purple. Color corresponds to the direction of expression change between the F_1_ crosses. All other significantly differentially expressed genes are shown in light gray. Points in dark gray are not significantly different

**Fig. 4:**
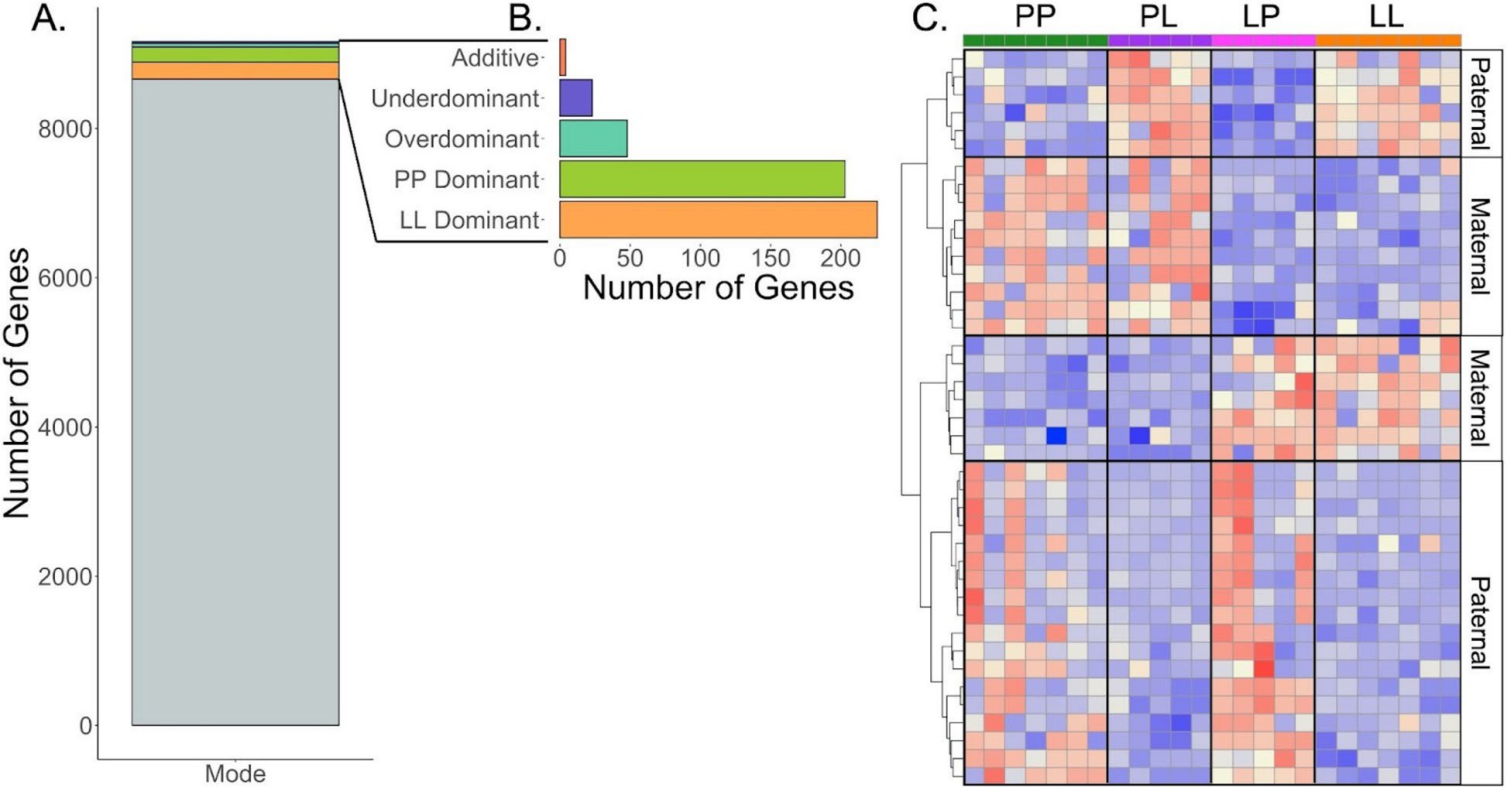
Mode of inheritance underlying expression divergence in eggs within a single species. **A** 8,722 genes are categorized as “conserved” (no expression level difference among parental or offspring’s eggs). **B** 523 of the remaining 1,155 genes that are differentially expressed between PP and LL eggs have been classified by their primary mode of inheritance. 102 genes are found to be differentially expressed between PL and LP. **C** A heatmap of genes with statistical support for parental effect direction. Relative gene expression is shown, with colors scaled per-gene. The expression direction (red is high expression and blue is low expression) for these genes matches the direction of either parental type. For example, if PP and PL have the same direction of expression (same color) this is a maternal effect, whereas if PL matches the direction of LL it is a paternal effect on expression. 24 genes are classified as primarily paternally inherited, and 17 as maternally inherited

Reciprocal crosses generated F_1_ females from PP and LL parents (Fig. 1). The F_1_ females were raised to maturity and oocyte mRNA was made into libraries in the same way as above. When the F_1_’s eggs are included in the PCA (Fig. 3a) we found they fall between the parentals on PC2, but the variance among F_1_ samples is high with some extreme expression values compared to the parents on PC1(Fig. 3a, b). Most transcripts (78%, 8,722 genes) have conserved expression levels among all eggs (PP, LL, and F_1_ expression levels are similar). Out of the 1,155 genes that are differentially expressed between PP and LL eggs, 504 of the genes could be confidently assigned a mode of inheritance based on expression in F_1_’s eggs (Fig. 4a, b) according to formal criteria (STable 3). 429 genes are dominant for one morph, 4 are additive, and 71 are over or underdominant. The remaining 651 differentially expressed genes either were not sufficiently sequenced in the F_1_ samples (uninformative) or have an ambiguous expression pattern likely due to high variation within F_1_ oocyte samples. The number of genes exhibiting dominant expression is almost even between P and L-allelic dominance, only slightly skewed in the L direction.

We compared the gene expression of the oocytes produced by reciprocal F_1_s (PL versus LP) to determine to what extent parent-of-origin effects influence maternally-loaded transcripts. Both F_1_ females are heterozygotes with respect to P and L alleles, so the only difference between PL and LP expression is the direction of the cross that gave rise to the mother (distinguishing if the allele is inherited from the maternal or paternal side). Genes that are differentially expressed between LP and PL are expressed in either the maternal or paternal direction, matching the expression of the P parent or the L parent. 102 genes are significantly differentially expressed between PL and LP (Fig. 3c), which accounts for 1.58% of the total number of genes expressed in oocytes. By comparing the expression of these genes back to PP and LL parental genotypes, we determine that 24 genes are exhibiting paternal effects, and 17 genes are exhibiting maternal effects, meaning they are overexpressed in the same direction (Fig. 4c,d). Gene annotations and group-mean expression values are given in STable 4. Briefly, this set of genes has a variety of functions, including zinc-finger proteins, several genes involved in various types of metabolism, and several cell-structure proteins. Additionally, we tested the possibility that one parents’ allele may be expressed preferentially even if there is no reciprocal differential expression. In our data this phenomenon occurs very infrequently: we found only three genes for which this is the case (one case of the maternal allele being expressed preferentially, and two cases for the paternal allele).

### Mode of regulatory change

**Fig. 5:**
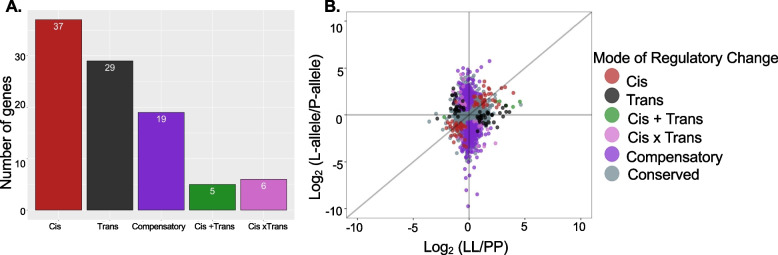
Regulatory architecture of differential expression in F_1_ oocytes. Both sets of F_1_s are analyzed together. Separate analysis of PL and LP showed similar results. **A** The number of genes in each category. **B** The distribution of differences between each morph’s gene expression versus differences in allelic expression in F_1_ oocytes. Each point represents one gene. Points falling close to the y = x diagonal axis have similar allelic expression differences in F_1_ oocytes compared to the ratio of gene expression found in PP and LL oocytes. Color shows the assigned mode of regulatory change

Many differentially expressed genes could be co-regulated by a few *trans*-acting factors in the maternal genome. To assign mode of regulation we used SNP differences in the transcripts of F_1_’s eggs to assign each read as either the P or L allele. This analysis was only able to assign parental origin to 21.6% of F_1_ reads as there are few differentiating SNPs in this intraspecific comparison. This should not bias results towards genes with one type of regulatory architecture because we do not expect the occurrence of polymorphic sites in a gene to correspond to a mode of regulatory change. We assigned the regulatory mode for 96 genes according to criteria shown in STable 5. We found that, contrary to expectations, genes whose expression could be explained by *cis*-regulatory differences between alleles was only slightly greater than the genes with *trans*-acting regulation (Fig. 5a). Some genes exhibit compensatory expression patterns (*n* = 19), and there are only a few *cis* + *trans* or of *cis* 𝚡 *trans* changes (Fig. 5a, STable 5). No clear pattern of bias towards either morph’s alleles emerges when comparing expression differences between parents to allelic expression patterns within F_1_ offspring (Fig. 5b), though it is clear that there are numerous genes with compensatory expression patterns which are mis-expressed in the F_1_s.

## Discussion

We investigated the changes in oocyte mRNA provisioning that accompany a common life-history transition in many animal taxa: increase in egg size. Using *S. benedicti*, we can investigate the regulatory architecture of changes in egg size and mRNA expression with F_1_ crosses. Females of the two developmental modes produce eggs with a large difference in volume, and while F_1_ females produce intermediate egg sizes, F_2_ females produce a variable range of egg sizes, meaning that egg size is a quantitative trait [32]. Evolutionary theory predicts that the switch from planktotrophic to lecithotrophic larvae initially involves an increase in egg size followed by adaptive changes to larval morphology [17, 51–54]. Despite the clear difference in egg size in *S. benedicti*, it was previously unknown whether the evolution of the larger LL egg included differences in maternal mRNA provisioning, and if these differences affect offspring ontogeny.

### Comparison of LL and PP egg gene expression

To investigate quantitative (expression presence/absence) and qualitative (differential expression) changes in mRNA provisioning, we first compared the mRNA expression between eggs of PP and LL mothers. We find that 43% (11,161 genes) of all genes (26,216 genes) in the *S. benedicti* genome are expressed in eggs of both morphs. This is more than in humans (36%; [55]), *Xenopus laevis* (20%; [56]), and coral *Montipora capitata* (12%; [57]) but fewer than some vertebrates: mice (63%; [56]), cows (44.7%; [56]), zebrafish (49%; [58]); and invertebrates: tunicate *Oikopleura dioica* (63%; [59]), *Drosophila* (65%; [60]). These studies represent a range of mRNA quantification techniques that likely lead to different estimates; however there is some conservation of genes that are maternally derived across all bilaterians [61].

Of the 11,161 genes expressed in *S. benedicti* eggs, 10.2% are differentially expressed between the PP and LL eggs, which is a large difference considering it is due to allelic variation within a single species. We are not aware of similar studies in other species with variable egg sizes for comparison. However, it is not unheard of for gene expression in eggs of the same species to vary considerably. Variation in maternal mRNA deposition can certainly occur within a species, particularly due to maternal condition or genotype. For example, in *Drosophila melanogaster* the difference in oocyte gene expression between extreme maternal environmental conditions (starved mothers fed 5% of control mothers’ diet) is ~ 1.8% [62, 63]. Another study, which sampled a much greater amount of genetic diversity, found maternal mRNA from embryos within populations of *D. melanogaster* to be much greater (40–60% of expressed genes differentially expressed; [64]. In urchins of the genus *Heliocidaris*, planktotrophic and lecithotrophic larvae originate from different species, which are five million years diverged. Through early development ~ 20% of their genes have different expression patterns, although this is not limited to the egg itself [65]. None of these studies are directly comparable to ours as they involve different treatments, developmental timing, and population structures. Therefore, whether the amount of differential expression we see in *S. benedicti* eggs is unusually high remains an open question.

We found 18 genes are exclusively expressed in eggs of one morph. While not a particularly large subset, these morph-specific genes indicate qualitative changes to the mRNA make-up of eggs in these morphs. Whether this set of genes has a substantial impact on development or subsequent gene expression remains to be seen. Nonetheless, these exclusive genes highlight the possibility that the maternally controlled initiation of development is functionally distinct between the morphs. With the other developmentally relevant DE transcripts, we have identified a set of genes that should be the target of future studies investigating maternally directed development in this species.

### Comparison of reciprocal F_1_ gene expression with LL and PP

Because F_1_ females are heterozygotes (PL or LP), and they produce intermediate sized eggs, we may expect to see additive inheritance resulting in intermediate expression levels. However, few genes have an additive mode of inheritance and dominance is more pervasive. Because the within-group variance of the F_1_’s egg samples is larger than the variance between PP and LL, and because F_1_ expression is often intermediate, there may be statistical power issues that limit our ability to infer additivity (Fig. 3a). (In this case, these genes would be classified as ‘ambiguous’). An alternative possibility is that the F_1_’s eggs would contain the same gene expression as one of their parents due to dominance. Dominance early in development may indicate that the genes of one morph are required before the maternal-zygotic transition. A third (38.9%) of differentially expressed genes are dominant, and there is almost equal dominance between P and L alleles with a slight increase in L-dominance. We also see low levels of misexpression (over or underdominance: 0.8%, Fig. 2b) in the F_1_’s eggs, which is consistent with misexpression scaling with evolutionary divergence of the parents [41].

Reciprocal crosses can also disentangle the contribution of each parent’s alleles to offspring phenotype [66–69]. Because we can make reciprocal crosses in *S. benedicti,* we are able to determine genes whose expression changes because of the parental background type alone: these are genes that have differential expression between LP and PL (Fig. 1). This analysis reveals the extent of differential expression and the particular genes that are impacted by genetic differences in the maternal background. Parent-of-origin effects have not previously been investigated during oogenesis. By comparing expression between the eggs of PL and LP females, we find that 41 genes exhibit parent-of-origin effects, which act in both the maternal and paternal direction. These expression differences occur when the F_1_ mother is producing her eggs, which means the transcript number an egg receives is due to the F_1_ mother’s parental cross direction. F_1_ females whose mother was PP provision their eggs differently than F_1_ females whose mother was LL, despite both F_1_ females having the same heterozygous genotype. (Although it is also possible that a minor amount of differentiation between F_1_ gene expression could be due to differences in the maternal mitochondrial haplotypes. Allele expression switching from maternal to paternal allele while keeping the total expression the same was extremely rare). Our results indicate that these parental-background effects persist and alter mRNA expression in eggs that make the next generation, which is an intergenerational effect on egg mRNA provisioning.

How do these parent-of-origin expression effects get passed to the next generation? Epigenetic modifications by maternally deposited mRNAs have been shown in vertebrates (zebrafish; [70]; mouse; [71]). In invertebrates, most studies to date have identified sncRNAs as the maternally inherited drivers of epigenetic change (C. elegans, [72–77], although it is unclear if this is a pervasive mechanism across invertebrate taxa. There is evidence for epigenetic modifications via histone variants in some species [78–80]. Perhaps the simplest mechanistic explanation would be gene regulation by methylation, but studies on invertebrates suggest methylation is unlikely to be modifying expression [81–83]. It is possible that, while these genes have significantly different expression patterns, they have no functional effect on development. This role of these parent-of-origin genes in development should be investigated in future studies. However, maternal and paternal effects are evident for some larval phenotypes in *S. benedicti* [32], suggesting that these maternally expressed differences could affect later developmental phenotypes. While the mechanism that causes parent-of-origin effects remains unknown, our results suggest there is some kind of epigenetic (intergenerational, G_maternal_ x G_zygotic_ interaction) regulation of reproduction.

### Mode of regulatory change

An advantage of our cross design is that we can determine the mode of regulation for differentially expressed genes. Typically regulatory analyses of *cis* and *trans* acting factors are conducted in hybrids [38–41, 63, 84], however, we were able to carry out these analyses in intraspecific comparisons with the caveat that there are fewer distinguishing polymorphisms between the alleles of the two parents. As such, we focus on the genes that are differentially expressed between the two morphs and therefore are not capturing the genetic architecture of genome-wide divergence between morphs, but rather the mode of regulation of genes that are differently expressed. Nonetheless, we are able to identify the regulatory mode of 6.7% of the differentially expressed genes.

It is possible that differences in embryonic gene expression are due to a small number of *trans*-acting factors that can act pleiotropically to change expression of multiple genes. This is a parsimonious explanation for multiple phenotypic differences arising from a small number of changes to the genome. In closely related species, modifications to *trans* regulatory elements are the main driver of expression divergence [84]. More distantly related species have more *cis*-acting regulatory differences, that presumably are selected for and individually modified over evolutionary time [38–41]. We find that expression differences in F_1_ oocytes are explained by an almost even combination of both *cis*-acting and *trans*-acting factors, and a few instances of interactions (*cis* 𝚡/ + *trans*). For genes that are not differentially expressed between the two morphs, we do detect many compensatory changes, where the total expression is the same across parents because *trans-*acting factors are compensating for *cis-*acting changes or vice versa, and therefore expression levels differ in the F_1_s. Theory predicts that this occurs under stabilizing selection for expression level [85], but over evolutionary time could lead to higher instances of misexpression.

## Conclusions

Our findings show that the mRNA provided to eggs of different sizes in *S. benedicti* is not simply a modified amount of the same maternal mRNAs. Maternal mRNA provisioning to eggs can vary significantly depending on the parents’ genotype. When comparing PP and LL eggs, we find significant expression differences for ~ 10% of the genes, and this difference is only due to allelic variation across parents. While changes in expression level make up the majority of the differences we see between morphs, there are also genes that exclusively appear in only one morph (only some of which are also expressed in the F_1_s: SFig. 2). A similar quantitative change in mRNA expression has been seen with egg size increases in other species such as echinoderms [59, 80], and may indicate a general phenomenon associated with increased egg size.

The regulatory analysis of reciprocal F_1_s demonstrates a complex genetic architecture in which maternal expression differences between the two morphs are due to both *trans* and *cis*-acting regulatory changes. Interestingly, this implies that numerous *cis* regulatory changes have evolved between these intraspecific types, which we may not expect in closely related species or populations. We also find differences in maternal expression in eggs can persist to the next generation, affecting how a mother packages mRNA to her own eggs. How these differences in mRNA egg expression affect downstream development remains to be determined, but this study shows that parental genetic effects on larval development could originate from morph-specific maternal provisioning.

## Methods

### Animal collection

We used lab-reared females descendent from two populations: PP worms are from Newark Bay Bayonne, New Jersey (40°41′11″N, 74°06′48W) and LL worms are from Long Beach, California (33.71°N, 118.28°W). These populations are consistent with those used in previous genetic studies [16, 31, 32, 86]. Females were reared in isolation such that there was no possibility of fertilization prior to sample collection. PP and LL individuals were reciprocally crossed to make F_1_ offspring PL and LP (maternal allele is listed first; Fig. 1). Female F_1_s were reared until gravid (also in isolation), when we extracted and measured their eggs.

### Oocyte RNA collection and library prep

Eggs were dissected from female bodies by mechanical isolation of tissues in ice cold PBS. Extracted oocytes were counted and moved immediately to Arcturus PicoPure (Ref: 12,204–01) RNA extraction buffer. The entire clutch of oocytes for a single-female was combined as one pooled sample. Seven PP, and seven LL clutches were used. A total of ten F_1_ oocyte pools were sampled, five from PL females, and five from LP females. A clutch of pooled oocytes is roughly the same amount of physical material regardless of maternal type, and the total RNA yield of clutches was similar in preliminary tests of the RNA extraction protocol as quantified by Qbit chemistry. Total input mRNA will not affect results of statistical tests in subsequent analyses due to established data normalization pipelines. Libraries were constructed with the NEB UltraII Stranded RNA library prep kit for Illumina. Libraries were sequenced on two lanes of 150 bp on the Illumina NovaSeq resulting in 80 million reads/library.

### Read quality trimming and alignment

We use a combination of TrimGalore (cutadapt [87] and FastP [88]) to trim ambiguous bases. To remove rRNA we used SortMeRNA [89], which identifies reads that map to a database of common eukaryotic rRNA sequences as well as annotated *S. benedicti* rRNA sequences. We aligned reads to the *S. benedicti* reference genome using HISAT2 [90] with strandedness, splice-site, and exon annotation guidance enabled using the default scoring parameters. SAM files were sorted by order of name using samtools sort, and feature-counting was done with HTSeq-count using the genome annotation file [86]. As the reference genome for *S. benedicti* is the planktotrophic morph, the final feature counts were averaged within each sample group to assess whether there was a significant bias towards this morph. We found PP samples had on average only 4% more of their total sequenced reads assigned to features than LL. This is a small difference which demonstrates no large mapping bias between the morphs. (STable 1).

### Normalization of F_1_ reads

When the F_1_ comparisons are incorporated in the analyses, all read counts were normalized together with the additional use of RUVg (RUVseq; [91]) where a set of a priori housekeeping genes is used to standardize across samples (STable 6). This was necessary as the F_1_ samples were sequenced separately from the planktotrophic and lecithotrophic samples. A normalization step based on housekeeping genes enables us to compare between the parental and F_1_ sample groups and remove variance added by batch effects from the two separate sequencing runs. This does not introduce bias due to egg size differences because the mean egg size of the parental samples is equal to the mean egg size of F_1_ samples, and because the selected housekeeping genes do not exhibit differential expression between the PP and LL eggs. This indicates that the expression of these housekeeping transcripts does not scale with egg size relative to other genes, making them a good reference for batch effects. After accounting for batch effects, variance stabilizing transformation was applied to counts in all four groups to make the PCA clustering, which is plotted for the first two principal components with ggplot2 (Fig. 2a) and a heatmap of the expression values using the R package ‘pheatmap’ (Fig. 2b).

### Differential expression

Feature counts from all samples were concatenated and normalized together with DESeq2’s median of ratios method [50]. This second sample normalization accounts for sequencing depth variance between each sample, independent of the sequencing batch. (Whereas the batch normalization step with RUVg permits comparisons between batches, the median of ratios normalization permits comparisons among all samples.) As part of the standard DESeq2 pipeline, *p*-values resulting from Wald tests were additionally corrected based on the Benjamini–Hochberg false discovery rate (FDR) algorithm to reduce the incidence of false positives for differential expression. Thresholds for significant differences were set as an FDR adjusted *p*-value of 0.05 or less, and an absolute fold change of more than 1.5x. Genes were considered very lowly expressed for a group if the group-mean normalized read count for a gene was below 10. If the other group’s mean normalized read count for those genes was above 150, then those genes were considered qualitatively differentially expressed. The significance level was adjusted for the analyses that include F_1_ oocyte RNAseq samples to accept FDR adjusted *p*-values of less than 0.10 in order to compensate for increased variability and mis-expression among the F_1_ samples. This adjustment allows us to classify more genes by their mode of inheritance and regulatory mode in subsequent analyses. It does not change the relative proportion of genes attributed to each category in any of those analyses.

### Mode of inheritance (Allele-specific effects)

To investigate the genetic basis of the expression differences found between PP and LL eggs (*n* = 7 of each), we made reciprocal F_1_ crosses (*n* = 5 in each cross direction; Fig. 1) and compared expression in their eggs back to PP and LL eggs. We assigned the primary mode of inheritance for each expressed gene using differential expression tests from DESeq2 according to established criteria (see STable 3; [41, 84]). First, genes with parent-of-origin specific expression (differences in expression between F_1_s: PL and LP) were removed. The remaining genes were classified as being additive, dominant for one haplotype, or mis-expressed in heterozygotes (over/under-dominant). For example, a P-dominant gene is expressed at the same level in PP, PL and LP eggs, but differentially expressed in LL eggs (FDR adjusted *p*-value 0.1). Genes whose expression level is intermediate in the F_1_s (PL or LP) compared to PP and LL eggs are classified as additive, and genes expressed in F_1_s (PL or LP) at lower or higher levels than both PP and LL eggs are classified as underdominant and overdominant respectively.

### Parent of origin effects

For those genes exhibiting differential expression between reciprocal F_1_s (*n* = 5 for each cross direction), the same classification was applied to each group independently, and the results were compared. In these cases, genes are considered maternal dominant if they were classified as P-dominant in PL eggs, but not in LP eggs, or L-dominant in LP eggs but not in PL eggs. Conversely, genes are considered paternal dominant if they were classified as L-dominant in PL eggs, but not in LP eggs, or P-dominant in LP eggs but not in PL eggs.

### Mode of regulatory change

Alleles from F_1_’s eggs were assigned to one of the two parents by identifying fixed SNPs within the transcripts. We used HyLiTE [92]; default parameters) to find SNPs and assign reads to the planktotrophic or lecithotrophic allele. To improve allele assignment rates, we included an additional 200 bp upstream and downstream of the input gene models to capture any reads which may align to untranslated regions not included in the annotations of coding sequences [84].

The per-gene categorization of regulatory mode followed established empirical methods [38, 39, 41, 84]. Only genes for which more than 20%, and no less than 10 total, of reads in the F_1_ samples could be assigned to a parent were considered. Three comparisons of each gene’s expression were made: First, we use genes that are differentially expressed between the parental morphs (PP and LL). Second, we calculate the allele-specific expression of each gene in the F_1_ (either PL or LP, *n* = 5 of each), using a negative binomial generalized linear model and Wald statistical tests using DESeq2 (v1.32.0). Third, we use a ratio of the differential expression of PP:LL alleles to the differential expression of LP:PL alleles. This analysis uses a significance level of 0.1 as its criterion for significance. We use DESeq2, using the design (~ W_1 + Geno * Ori) where W_1 is the normalization factor returned by RUVg, Geno identifies reads as either a P or L allele, and Ori identifies the reads as originating from the parentals or F_1_ samples. Based on the results of the three above comparisons, DE genes were categorized as either in “cis”, “trans”, “cis + trans”, or “cis * trans” [38, 39, 41, 84]. Further explanation of cis/trans categories in STable 5.

### Functional annotations

Previously established genome annotations [86] were improved by performing a BLASTx search against the complete UniProt/SwissProt protein databases, adding any annotations that had an e-value less than 10^–30^. Gene functional information was retrieved from the UniProtKB database. We used gene ontology (GO) information (SFig. 1) but did not test for term enrichment because we could not assign terms to the majority of the genes in our dataset.

## Supplementary Information

**Additional file 1:** **Supplement Table 1.** Sequencing and read processing results for all libraries. **Supplement Table 2.** Genes exclusive to one group: mean counts and annotations. **Supplement Table 3.** Criteria for mode of inheritance assignments. **Supplement Table 4.** Annotation of differentially expressed genes with parental effects. **Supplement Table 5.** Criteria for regulatory mode assignments. **Supplement Table 6.** Selected Housekeeping genes (for RUVg). **Supplement Figure 1.** REVIGO tree-map of GO terms associated with differentially expressed genes between PP and LL eggs. **Supplement Figure 2.** Differences in F1 egg expression for genes exclusive to LL or PP eggs. **Supplement Figure 3.** Shared gene expression across all groups.

## Data Availability

The datasets generated during the current study are available in the NCBI GEO repository, ACCESSION NUMBER: GSE225230. Updated functional annotations are submitted to NCBI. All analyses and datasets are available in the supplemental files, which contain input data and a Rmarkdown file. The GitHub repository for this project is: https://github.com/NathanDHarry/Harry-Zakas2023
