# Supplementary material for: Maternal patterns of inheritance alter transcript expression in eggs
Source: BMC Genomics. 2023 Apr 10;24:191. doi: 10.1186/s12864-023-09291-8 (PMC10084599; doi:10.1186/s12864-023-09291-8)

**Supplement**

*Supplement Table 1. Sequencing and read processing results for all libraries.*


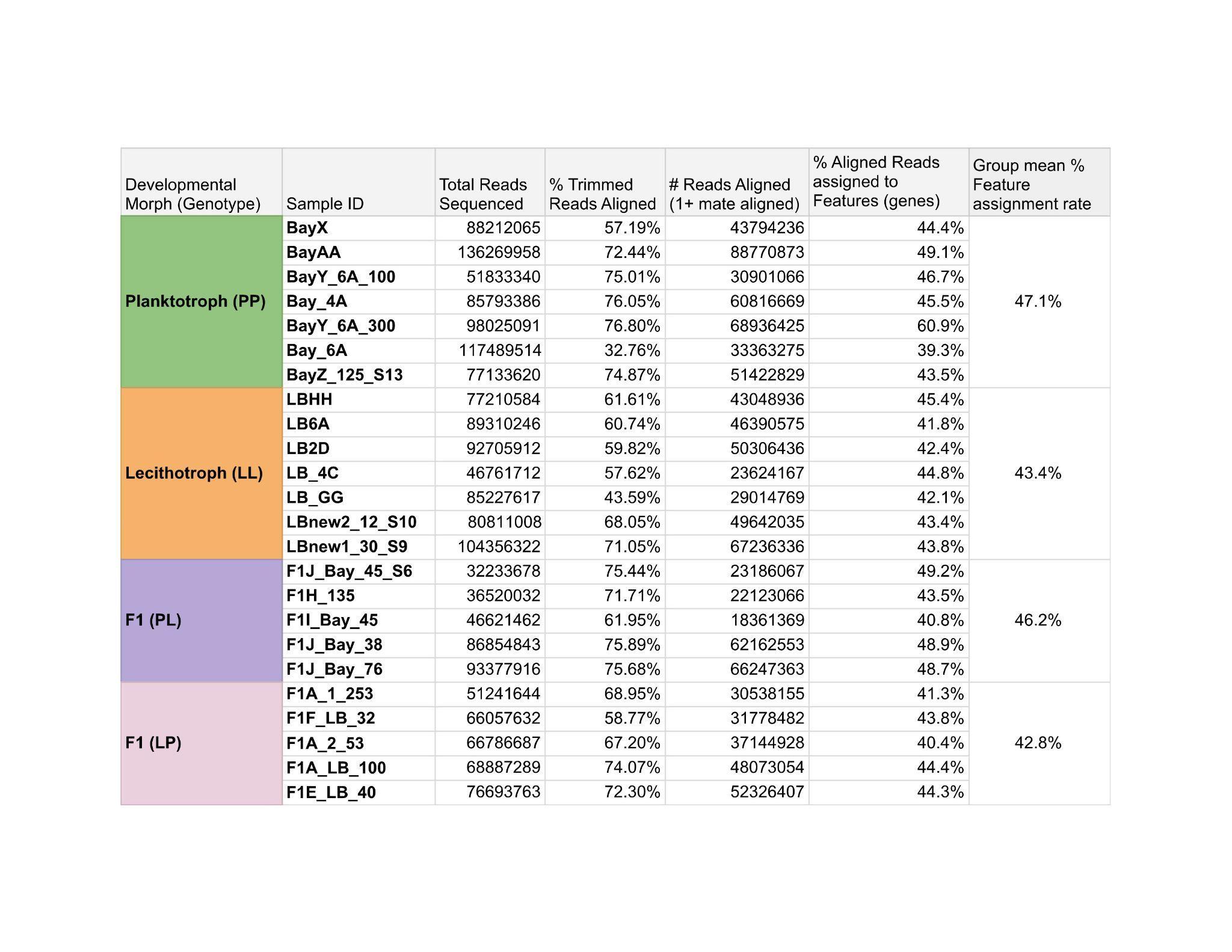


*Supplement Table 2. Genes exclusive to one group: mean counts and annotations*


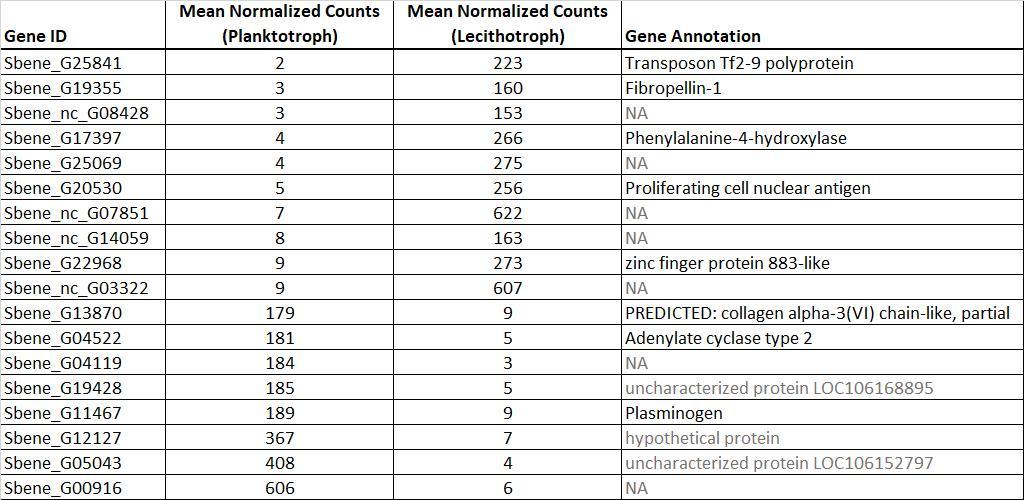


*Supplement Table 3: Criteria for mode of inheritance assignments*


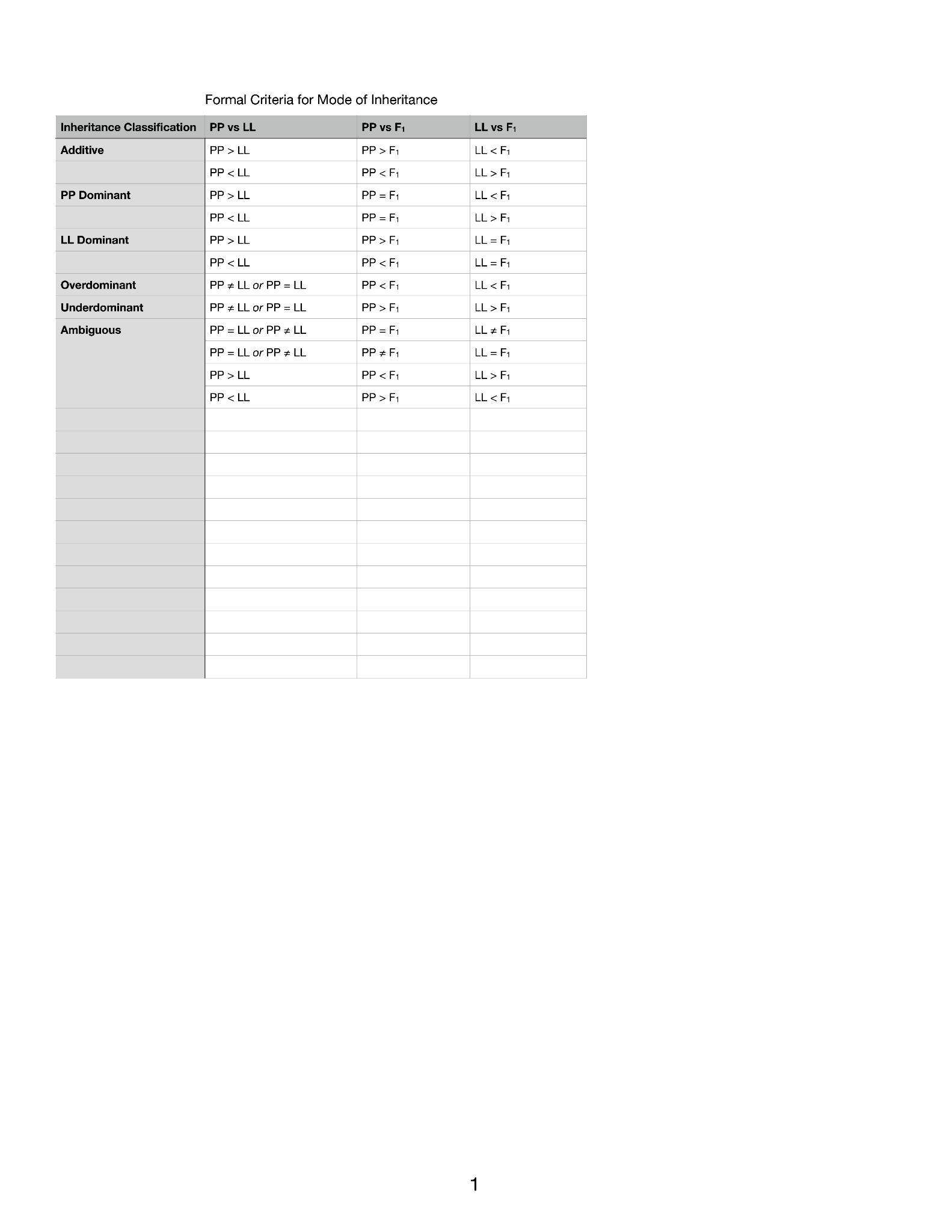


*Supplement Table 4: Annotation of differentially expressed genes with parental effects*


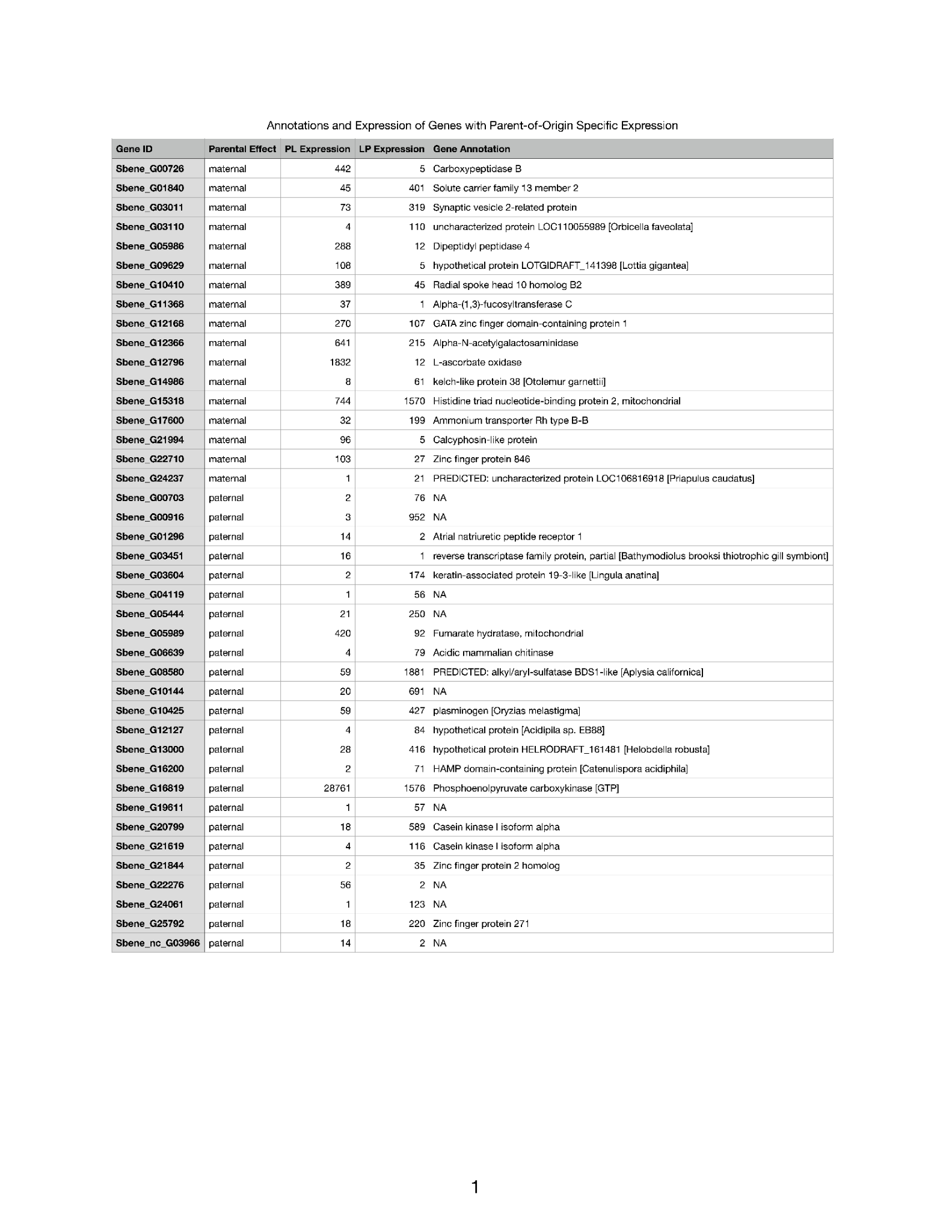


*Supplement Table 5: Criteria for regulatory mode assignments*


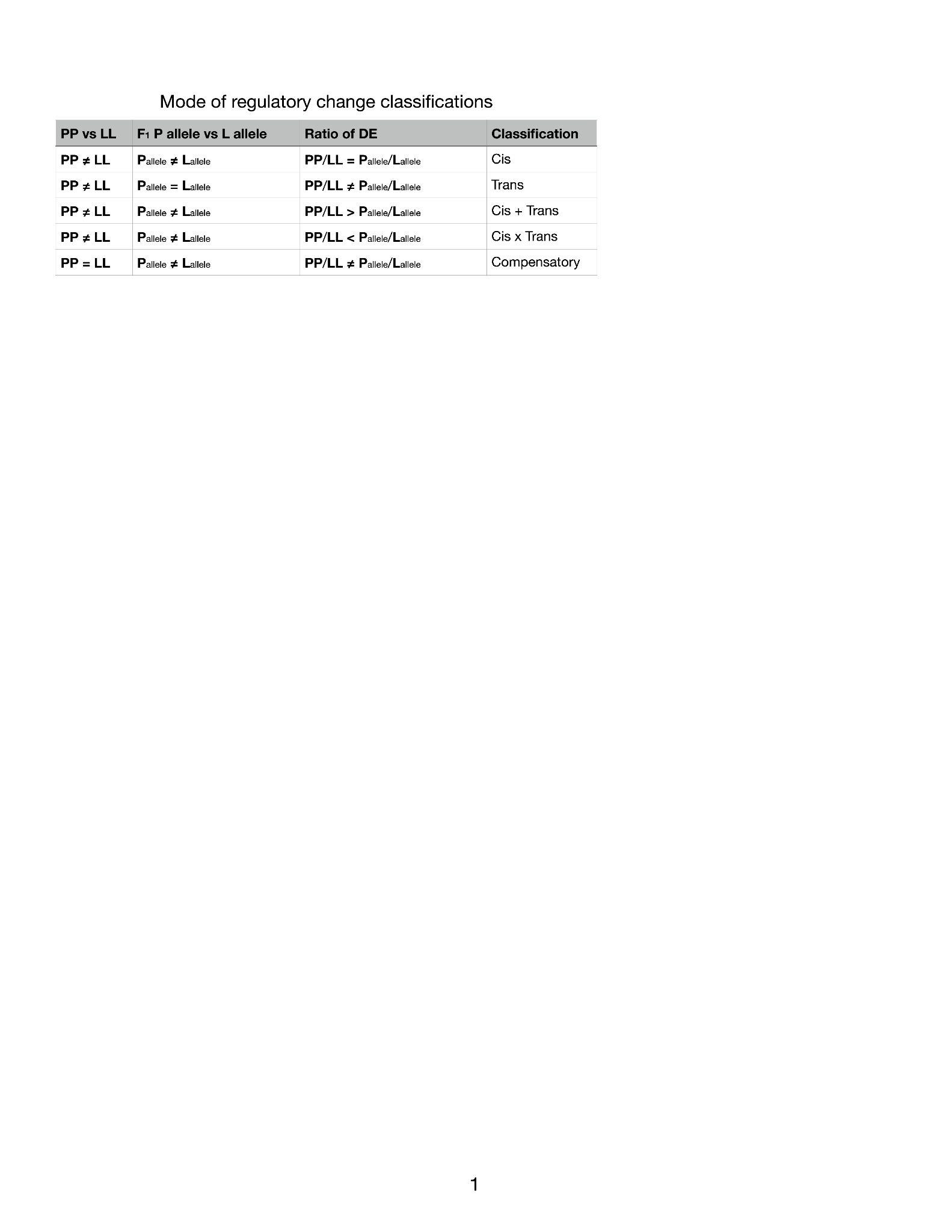


*Supplement Table 6: Selected Housekeeping genes (for RUVg)*
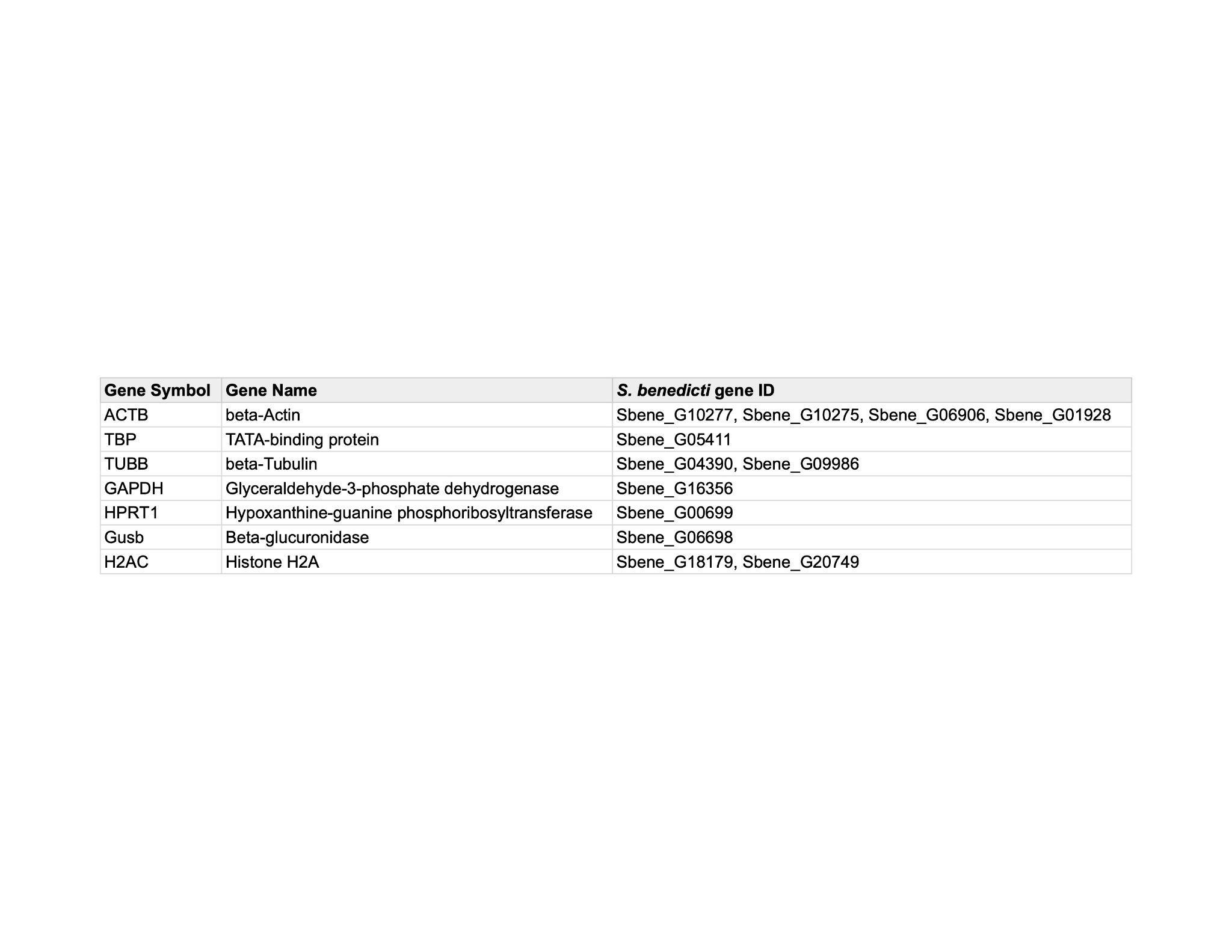


S*upplement Figure 1: REVIGO tree-map of GO terms associated with differentially expressed genes between PP and LL eggs*

**
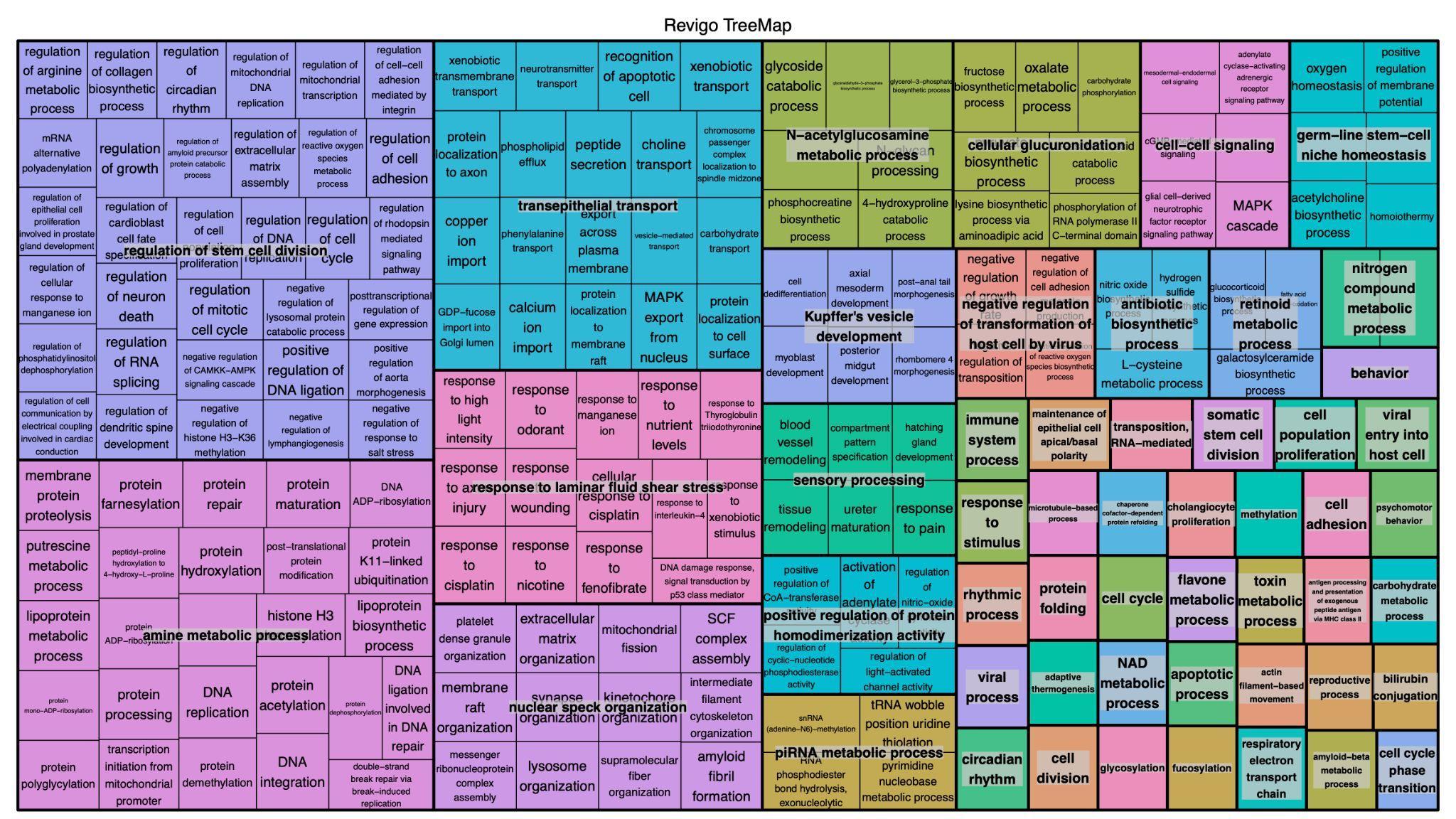
**

*Supplement Figure 2. F_1_ egg expression for genes exclusive to LL or PP eggs. (A) Gene expression in F_1_s when the transcript is expressed only in PP eggs (n= 8). (B) Gene expression in F_1_s when the transcript is expressed only in LL eggs (n=10). F_1_ expression values for PL and LP are combined for genes in both figures with the exception of one gene in A for which the expression of the reciprocal F_1_s varied greatly between parental backgrounds in a clearly paternal direction. One PP-exclusive gene (Sbene_G00916) is highly expressed in the F_1_’s eggs, and among the LL-exclusive genes, only one is highly expressed in the F_1_’s eggs (Sbene_G25841).*

A. B.


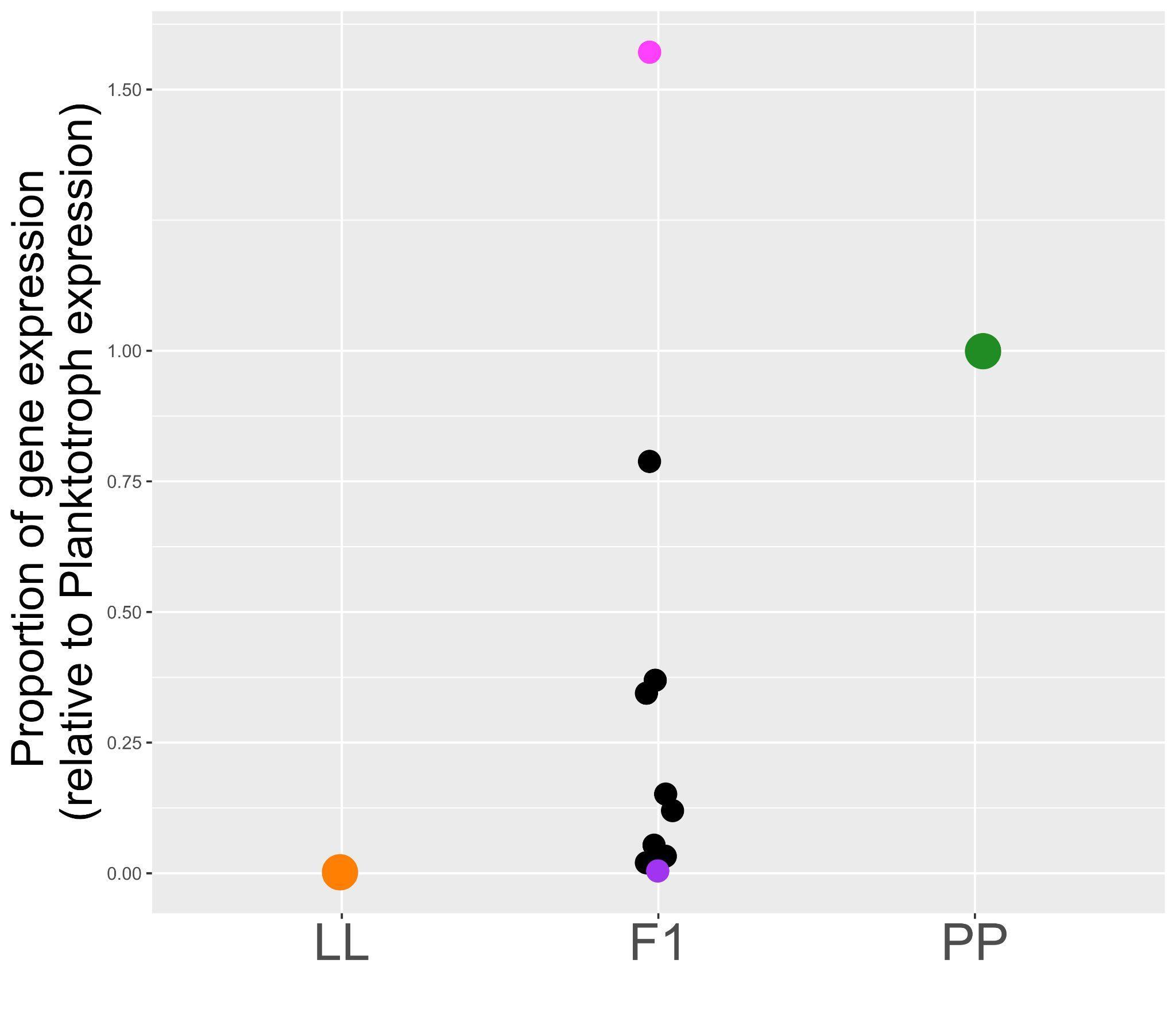

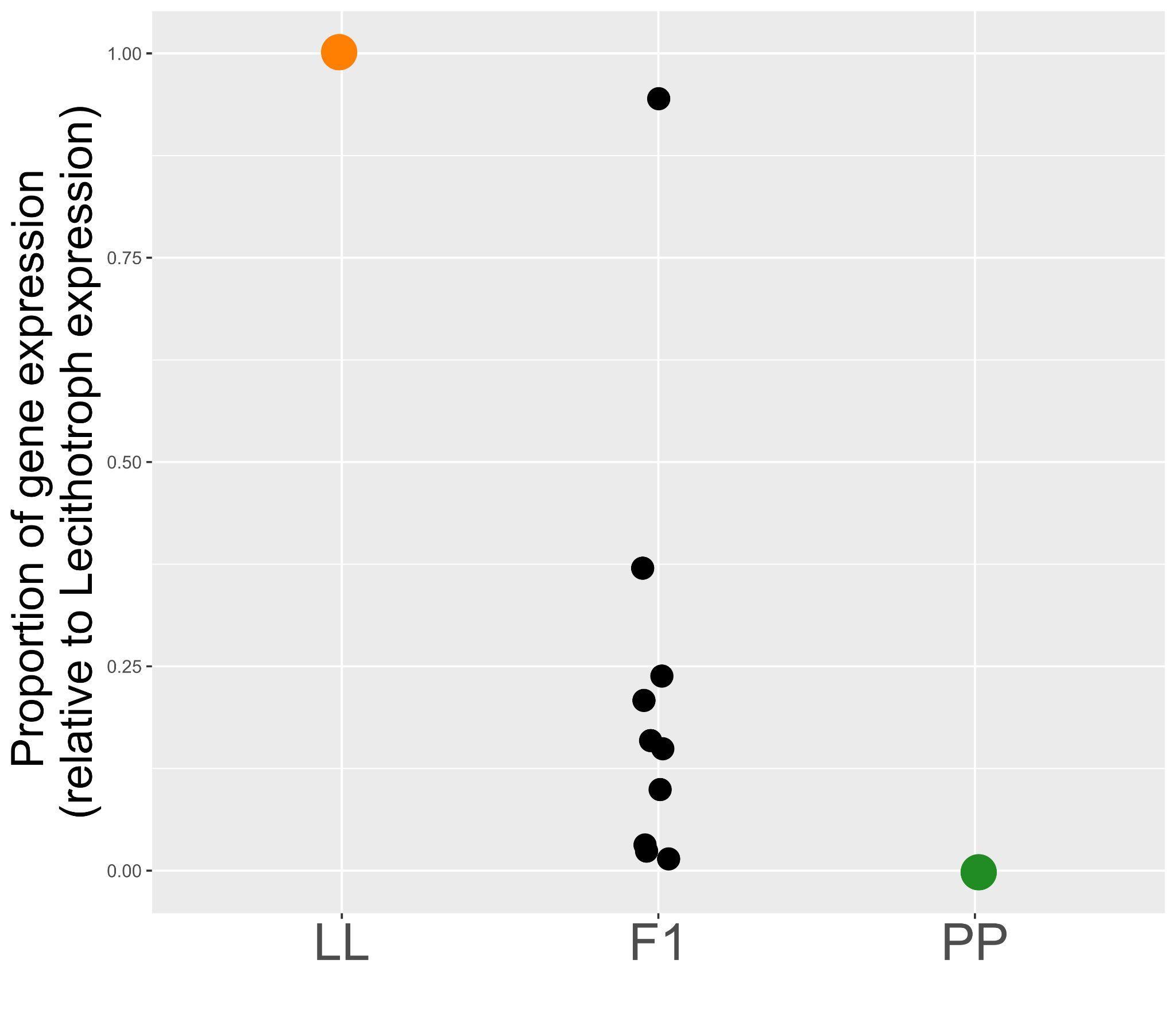


*Supplement Figure 3. Shared gene expression across all groups. 38 genes are differentially expressed between the F_1_s are also differentially expressed between the parent groups PP and LL. 64 genes are differentially expressed between the F_1_s that are not differentially expressed in the parents.*


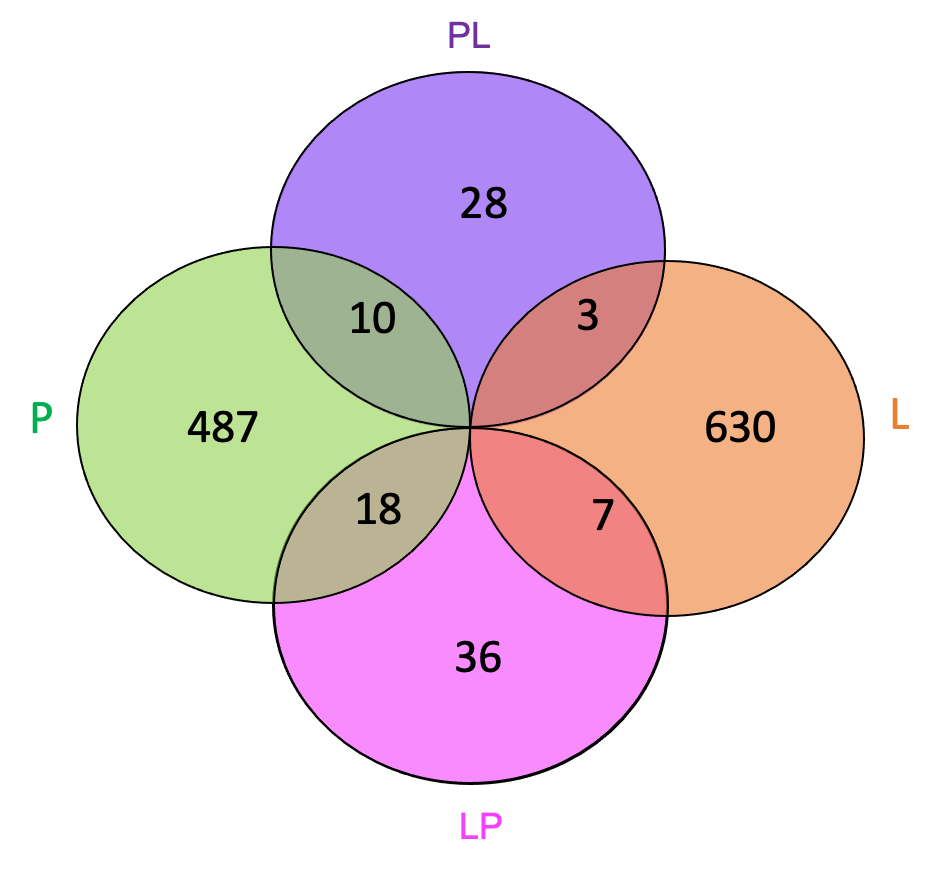

Supplement: Supplementary file 1 — Additional file 1: Supplement Table 1. Sequencing and read processing results for all libraries. Supplement Table 2. Genes exclusive to one group: mean counts and annotations. Supplement Table 3. Criteria for mode of inheritance assignments. Supplement Table 4. Annotation of differentially expressed genes with parental effects. Supplement Table 5. Criteria for regulatory mode assignments. Supplement Table 6. Selected Housekeeping genes (for RUVg). Supplement Figure 1. REVIGO tree-map of GO terms associated with differentially expressed genes between PP and LL eggs. Supplement Figure 2. Differences in F1 egg expression for genes exclusive to LL or PP eggs. Supplement Figure 3. Shared gene expression across all groups. [file 12864_2023_9291_MOESM1_ESM.docx]
